# Case Report: Ivonescimab in EGFR-mutant lung cancer with baseline malignant pleural effusion and acquired complex resistance

**DOI:** 10.3389/fimmu.2025.1725067

**Published:** 2025-12-17

**Authors:** Zeming Mo, Honglian Jiang, Lei Zhou, Qiaoya Ren, Gang Shen, Lie Li, Xiaobin Jian, Hua Yang, Yuju Bai, Mi Meng, Sisi He

**Affiliations:** 1 Department of Oncology, Key Laboratory for Cancer Prevention and Treatment of Guizhou Province, The Second Affiliated Hospital of Zunyi Medical University, Zunyi, Guizhou, China; 2 Department of Nephrology, The People’s Hospital of Qiannan, Duyun, Guizhou, China; 3 Department of Pathology, The Second Affiliated Hospital of Zunyi Medical University, Zunyi, Guizhou, China

**Keywords:** case report, EGFR mutation, ivonescimab, lung adenocarcinoma, malignant pleural effusion

## Abstract

**Background:**

Patients with epidermal growth factor receptor (EGFR)-mutant lung adenocarcinoma (LUAD) presenting with malignant pleural effusion (MPE) at diagnosis have a poor prognosis. Options are limited after EGFR-TKI resistance. Ivonescimab, a PD-1/VEGFA bispecific antibody, is effective in advanced non-small cell lung cancer, but its efficacy in patients with baseline MPE and complex acquired resistance remains unclear.

**Case presentation:**

A 55-year-old man was diagnosed with LUAD and significant MPE, featuring an EGFR exon 19 deletion and over 20 co-mutations. The patient eventually developed resistance to both first-line gefitinib and later almonertinib. Repeat genomic testing of pleural fluid upon progression revealed persistent alterations in 12 genes, including the original EGFR sensitizing mutation, TP53, AKT2, RARA, and SETD2, alongside newly acquired mutations in CHEK1, CUL3, DNMT1, HMCN1, and TBX3, and RB1 copy number loss, in the absence of typical resistance mechanisms such as T790M or MET amplification or histologic transformation to small cell lung cancer. PD-L1 expression on the effusion cell blocks was positive (TPS 40%, CPS 41). He received ivonescimab monotherapy, achieving disease control for nearly 5 months before transitioning to ivonescimab plus pemetrexed with continued benefit and a manageable safety profile.

**Conclusion:**

This case illustrates the potential benefit of ivonescimab in patients with EGFR-mutant LUAD and baseline MPE who develop complex, non-canonical resistance to EGFR-TKIs. These findings support further clinical evaluation of ivonescimab in this poor-prognosis subgroup and highlight the importance of repeated molecular profiling in guiding treatment strategy.

## Introduction

Lung cancer remains the leading cause of cancer-related incidence and mortality worldwide (1), with pathological types commonly classified into two major categories: small cell lung cancer (SCLC) (2) and non-small cell lung cancer (NSCLC) (3). NSCLC accounts for over 80% of lung cancer cases (4). Precision medicine for NSCLC, driven by advances in and the clinical application of diverse targeted therapies and immunotherapies, now encompasses the management of malignant pleural effusion (MPE) (5, 6). Approximately 40% of patients with lung cancer develop pleural effusions (7), and the proportion of patients with MPE approaches 50% even among those with the adenocarcinoma subtype (8). MPE is a well-established predictor of poor prognosis, particularly when present at initial diagnosis (7–9). Moreover, evidence suggests that patients with EGFR-mutant lung cancer have a higher incidence of MPE than those with wild-type EGFR (10–12). For patients with EGFR-mutated NSCLC who develop MPE, treatment strategies involving third-generation epidermal growth factor receptor tyrosine kinase inhibitors (EGFR-TKIs) or sequential first- followed by third-generation EGFR-TKIs have been shown to further extend survival (10). Nonetheless, acquired resistance to EGFR-TKIs remains a major clinical challenge (13). Collective data from pivotal trials demonstrate that the median progression-free survival (PFS) with approved third-generation EGFR-TKIs remains under two years, underscoring the fundamental limitation of ultimately inevitable treatment resistance (14). Upon disease progression, the prognosis for patients with MPE deteriorates significantly (15, 16).

Although intrapleural perfusion with chemotherapy or antiangiogenic agents is commonly used for local control of MPE (17), adding such perfusion to ongoing EGFR-TKI therapy after effusion recurrence may not improve survival and can increase treatment-related toxicity (18). For instance, in patients with acquired T790M mutation, combination therapy with an EGFR-TKI and bevacizumab was associated with a significantly longer PFS compared to bevacizumab plus chemotherapy (median PFS 6.9 vs. 4.6 months; P = 0.022), whereas T790M-negative patients derived less benefit (15). Moreover, the development of MPE upon gradual progression during EGFR-TKI therapy is a negative predictor of outcomes for subsequent treatment with EGFR-TKI plus bevacizumab (19).

Tumor cells within MPE can express programmed cell death protein 1 (PD-L1), and patients with PD-L1 positive (TPS ≥1%) effusions may derive better clinical benefit from immune checkpoint inhibitors (ICIs) than those with PD-L1 negative disease (20). Nevertheless, the presence of MPE is itself associated with worse outcomes following ICI therapy in LUAD, even among patients with PD-L1 positive (TPS>1) tumors (21). Furthermore, one study reported no significant difference in overall survival (OS) between EGFR/ALK-positive patients with MPE exhibiting PD-L1 TPS <50% versus ≥50% (21.0 vs. 20.5 months; P = 0.21) (22). These conflicting results highlight the uncertainty regarding the efficacy of ICIs in EGFR-mutant patients with MPE after EGFR-TKI failure.

Ivonescimab is a novel bispecific antibody targeting both programmed death-1 (PD-1) and vascular endothelial growth factor (VEGF)-A, designed to simultaneously inhibit immune checkpoints and block angiogenesis. In patients with advanced EGFR-mutated non-small cell lung cancer (NSCLC) that progressed on EGFR-TKIs, ivonescimab combined with chemotherapy had demonstrated longer PFS compared to regimens such as chemotherapy plus amivantamab and lazertinib or chemotherapy plus immunotherapy and bevacizumab (23). In May 2024, ivonescimab in combination with pemetrexed and carboplatin received its first approval in China for the treatment of EGFR-mutant locally advanced or metastatic non-squamous NSCLC following EGFR-TKI progression (24). Additionally, in previously untreated, PD-L1 positive advanced NSCLC, ivonescimab monotherapy resulted in significantly longer PFS compared to pembrolizumab (25), leading to its approval by the NMPA in April 2025 for first-line treatment of PD-L1 positive (TPS ≥1%) disease. These advances underscore the transformative potential of ivonescimab in managing EGFR-TKI–resistant and/or PD-L1–positive NSCLC.

However, the efficacy of ivonescimab in managing EGFR-TKI–resistant NSCLC with MPE, particularly in cases exhibiting complex molecular features such as high PD-L1 expression and numerous co-occurring genomic alterations, remains unestablished. Here, we present a unique case of a patient with baseline MPE and a high-complexity tumor mutational profile who, after developing resistance to sequential first- and third-generation EGFR-TKIs, achieved sustained disease control with ivonescimab-based therapy. This case provides critical early insight into the potential role of dual PD-1 and VEGFA blockade in overcoming resistance in molecularly complex NSCLC with MPE and underscores the need for further investigation into biomarker-driven treatment strategies for this challenging patient population.

## Case report

A 55-year-old male presented in March 2020 with a 3-month history of irritative cough and acute dyspnea. Chest CT revealed a left hilar mass with large pleural effusion and atelectasis. Following therapeutic thoracentesis, bronchoscopic biopsy on April 7, 2020 confirmed adenocarcinoma. Cytologic examination of pleural fluid revealed atypical cells. Comprehensive next-generation sequencing (NGS) (425+ gene panel; Genecast Biotech Ltd, China) of pleural fluid, FFPE tissue, and peripheral blood identified an EGFR exon 19 deletion (p.E746_A750delELREA) with allele frequencies of 2.51% in pleural fluid and 18.92% in tissue. Testing detected 24 additional somatic alterations (including AKT2, BRCA1, MYC, and TP53) (**Supplementary Table 1**), with no mutations in matched blood. The tumor was microsatellite-stable with high tumor mutational burden (TMB). Final staging was cT2aN2M1a (Stage IVA).

**Figure 1:**
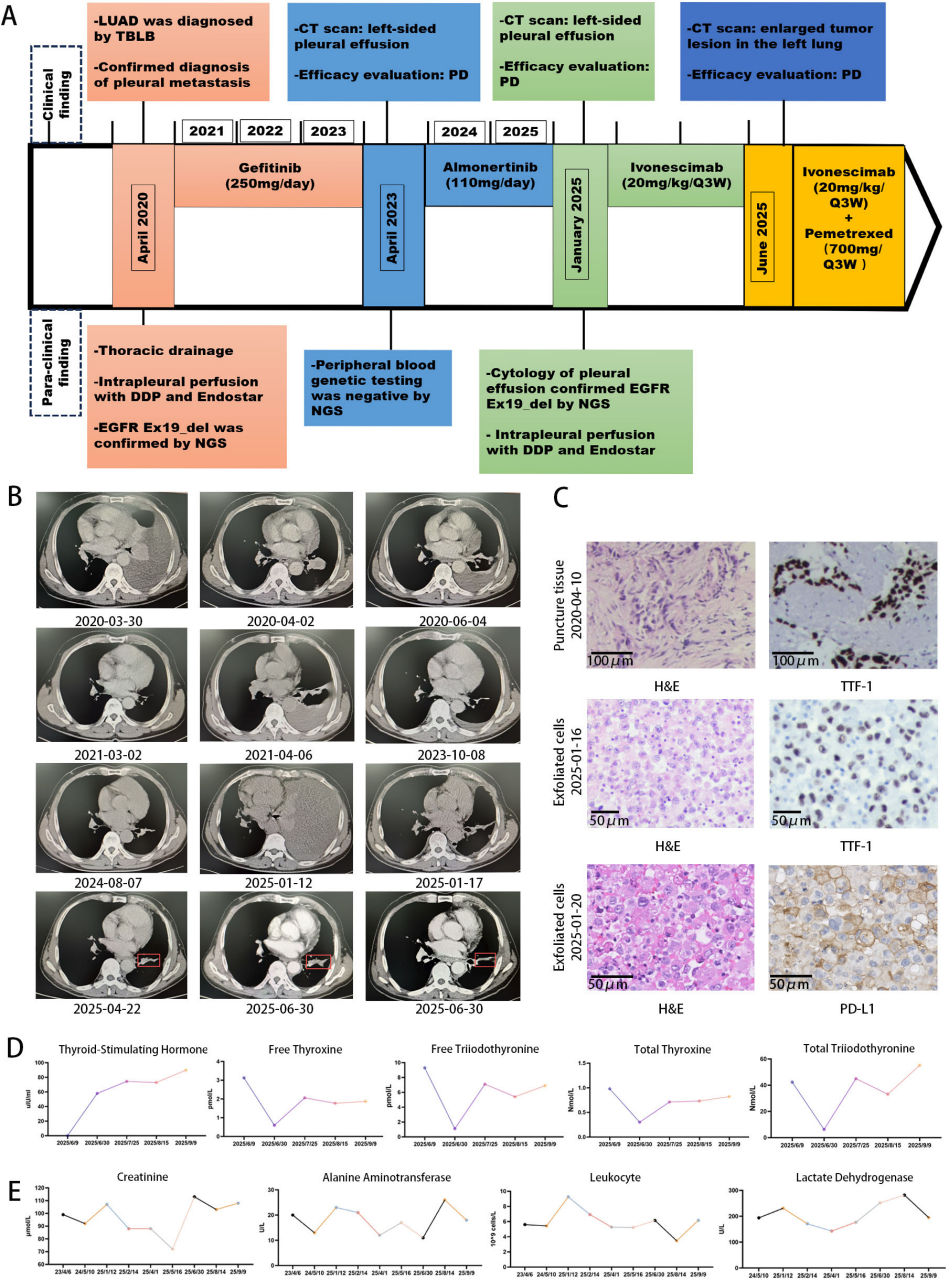
Treatment timeline, disease course, and adverse events. **(A)** Diagnostic and therapeutic timeline for the patient. **(B)** Representative serial chest CT images comparing the dynamic changes of the primary lung lesion and malignant pleural effusion. The red boxes highlight the target lesions evaluated for treatment response before and after therapy with ivonescimab plus pemetrexed. **(C)** Histopathological and immunohistochemical (IHC) analyses. Top panel: H&E staining and IHC (original magnification × 40) for TTF-1 of the initial diagnostic biopsy (FFPE tissue). Middle panel: H&E staining and IHC for TTF-1 of pleural effusion cytology samples after disease progression on almonertinib. Bottom panel: H&E staining and IHC (original magnification × 200) for PD-L1 (clone 22C3) on cell block sections derived from malignant pleural effusion after almonertinib progression (Tumor Proportion Score, TPS = 40%). **(D)** Line graph depicting the trend of serum thyroid function biomarkers over time before and after the initiation of combination therapy with ivonescimab and pemetrexed. **(E)** Line graph showing the trends of key laboratory safety parameters (creatinine, alanine aminotransferase, leukocyte count, and lactate dehydrogenase) over time.

The patient received two cycles of intrapleural cisplatin (April 2020) followed by recombinant human endostatin, concurrently initiating gefitinib (250 mg orally, once daily; **Figure 1A**). Surveillance CT on April 6, 2023 showed new left pleural effusion, indicating progression (**Figure 1B**). Repeat liquid biopsy (peripheral blood) NGS (identical panel) revealed no detectable mutations. Therapy was switched to almonertinib (110 mg orally, once daily), with subsequent imaging on October 8, 2023 demonstrating marked effusion reduction, indicating treatment response.

On January 12, 2025, CT showed recurrent large left pleural effusion with atelectasis, indicating progression. Repeated NGS of pleural fluid confirmed persistence of the EGFR exon 19 deletion (allele frequency 37.3%), along with mutations in AKT2, EGFR, RARA, SETD2, and TP53, among others (**Supplementary Table 1**), and newly acquired mutations in CHEK1, CUL3, DNMT1, HMCN1, and TBX3. PD-L1 expression on MPE cell blocks was high (TPS 40%; CPS 41) (**Figure 1C**), with low TMB. The patient received intrapleural recombinant human endostatin (a fixed dose of 30 mg) on January 16, 2025, and cisplatin (a fixed dose of 30 mg) on January 20 and 23, 2025 for symptomatic control. Given progression on almonertinib and PD-L1 positivity, combination therapy with ivonescimab and chemotherapy was recommended. However, due to concerns regarding toxicity, the patient elected to proceed with ivonescimab monotherapy. He received 7 cycles of ivonescimab (20 mg/kg IV Q3W) from January to June 2025, achieving stable disease. Subsequent CT on June 30, 2025, showed an interval increase in both the caliper and extent of a pre-existing strand-like parenchymal opacity in the left lung, indicating progression. The patient then consented to combined therapy, receiving 3 cycles of ivonescimab (20 mg/kg IV Q3W) plus pemetrexed (500mg/m² IV Q3W). The most recent CT in August 2025 showed stable disease, with no new metastases on brain MRI or abdominal CT. The patient remains on combination therapy.

Ivonescimab monotherapy was well-tolerated without significant toxicities. Following initiation of combination therapy, he developed asymptomatic hypothyroidism (Grade 2, CTCAE v5.0) (**Figure 1D**), managed successfully with levothyroxine without treatment interruption. No other significant treatment-related adverse events were observed in leukocyte count, liver, or renal function throughout the treatment course, demonstrating a favorable safety profile (**Figure 1E**).

## Discussion

This case demonstrates that ivonescimab can induce meaningful disease control in a patient with EGFR-mutant LUAD, MPE, and a highly complex genomic profile (>20 mutations, PD-L1 TPS 40%) following resistance to sequential EGFR-TKIs. Therapy was well-tolerated overall. Previous studies have suggested a negative correlation between PD-L1 expression in malignant effusions—particularly on immune cells—and patient prognosis (26–28). Furthermore, VEGF within malignant serous cavities is a key driver of effusion formation and is significantly associated with poorer outcomes in lung cancer (29). Given that MPE constitutes an immunosuppressive microenvironment potentially modulated by VEGF (30, 31), the dual targeting of VEGFA and PD-1 by ivonescimab may produce synergistic effects, reversing local immune suppression.

This case utilized two anti-angiogenic drugs: recombinant human endostatin and ivonescimab. Historically, agents like recombinant human endostatin and bevacizumab have been used to manage NSCLC-related MPE. As a cornerstone anti-angiogenic therapy, bevacizumab is frequently used for MPE, and intrapleural infusion may be more effective and safer than intravenous delivery (32). A meta-analysis confirmed that intracavitary bevacizumab plus cisplatin yields a superior overall response rate compared to cisplatin monotherapy, without a significant rise in toxicity (33). Regarding combination strategies, bevacizumab plus EGFR-TKI improves PFS versus bevacizumab plus chemotherapy in T790M-positive patients with MPE, whereas no such benefit was seen in T790M-negative patients (15). Importantly, MPE is an independent adverse prognostic factor for EGFR-mutant patients with gradual progression receiving original TKI plus bevacizumab (19), and bevacizumab combined with osimertinib may not extend PFS in treatment-naïve patients with MPE (34). Collectively, these findings underscore the need for more effective anti-angiogenic strategies, particularly in T790M-negative cases like ours.

Elevated levels of VEGF and endostatin in pleural effusion are positively correlated with poor prognosis in lung cancer (35). Recombinant human endostatin has been validated to inhibit both angiogenesis and lymphangiogenesis in MPE (36). Consistent with this, intrapleural administration of recombinant human endostatin combined with cisplatin has demonstrated higher MPE control rates and improved quality of life compared to cisplatin alone (37–39). Based on this evidence, the combination was employed as a local therapy for MPE in the present case.

However, a well-recognized limitation of anti-angiogenic therapy is the dissociation between PFS and OS, wherein PFS improvement does not reliably translate into an OS benefit. This phenomenon is frequently observed with intravenous anti-angiogenic agents in patients with acquired EGFR-TKI resistance (40–42). A potential strategy to overcome this limitation may lie in combining anti-angiogenic agents with anti-PD-1/PD-L1 inhibitors and chemotherapy. Several randomized controlled trials indicate that such triple-therapy regimens improve OS in advanced NSCLC compared to anti-angiogenics plus chemotherapy or immunotherapy plus chemotherapy (43–45). A retrospective analysis further supports the efficacy of the atezolizumab plus bevacizumab, carboplatin, and paclitaxel (ABCP) regimen in NSCLC patients with MPE (46). Nevertheless, the OS benefit of this triple combination remains controversial specifically in the EGFR-TKI-resistant population (40, 47, 48).

The HARMONi-A study established that ivonescimab combined with chemotherapy significantly improved both PFS and OS, versus chemotherapy alone, in EGFR-positive NSCLC patients after progression on a third-generation EGFR-TKI, while maintaining a manageable safety profile (49, 50). The high concordance of PD-L1 expression between histologic specimens and MPE cell blocks (51) further implies that ivonescimab’s activity may extend to primary and occult metastatic lesions. Collectively, these factors likely contributed to the nearly five months of disease control and only slow progression of the primary lesion observed with ivonescimab monotherapy in this case. Nevertheless, it is premature to conclude that ivonescimab overcomes the PFS-OS dissociation typical of traditional anti-angiogenic agents in patients with MPE. This hypothesis requires validation through larger real-world datasets and dedicated randomized controlled trials.

The high PD-L1 expression observed in the malignant pleural effusion cells predominantly originated from the tumor cells themselves, as indicated by the near-identical TPS and CPS scores. This finding strengthens the rationale for PD-1/PD-L1 axis inhibition in this patient, as the target ligand is abundantly present on the malignant cells. Although the prognostic implications of PD-L1 expression specifically on tumor cells versus immune cells within MPE remain unresolved, this case demonstrates that ivonescimab can yield substantial clinical benefit in a patient with tumor cell-positive PD-L1 expression. High PD-L1 expression (TPS ≥1%) on MPE cell blocks may thus represent a potential positive predictive biomarker for ivonescimab response.

Interestingly, the onset of hypothyroidism (Grade 2) occurred only after initiating combination therapy with ivonescimab and pemetrexed, not during ivonescimab monotherapy. This pattern of toxicity may represent a first clinical observation specific to ivonescimab-based combinations. Previous studies have reported an increased incidence of hypothyroidism when ICIs are combined with chemotherapy with or without antiangiogenic agents in chemotherapy-naïve, EGFR-TKI-resistant NSCLC patients (52); however, the synergistic mechanism underlying this endocrine immune-related adverse event remains unclear.

**Figure 2:**
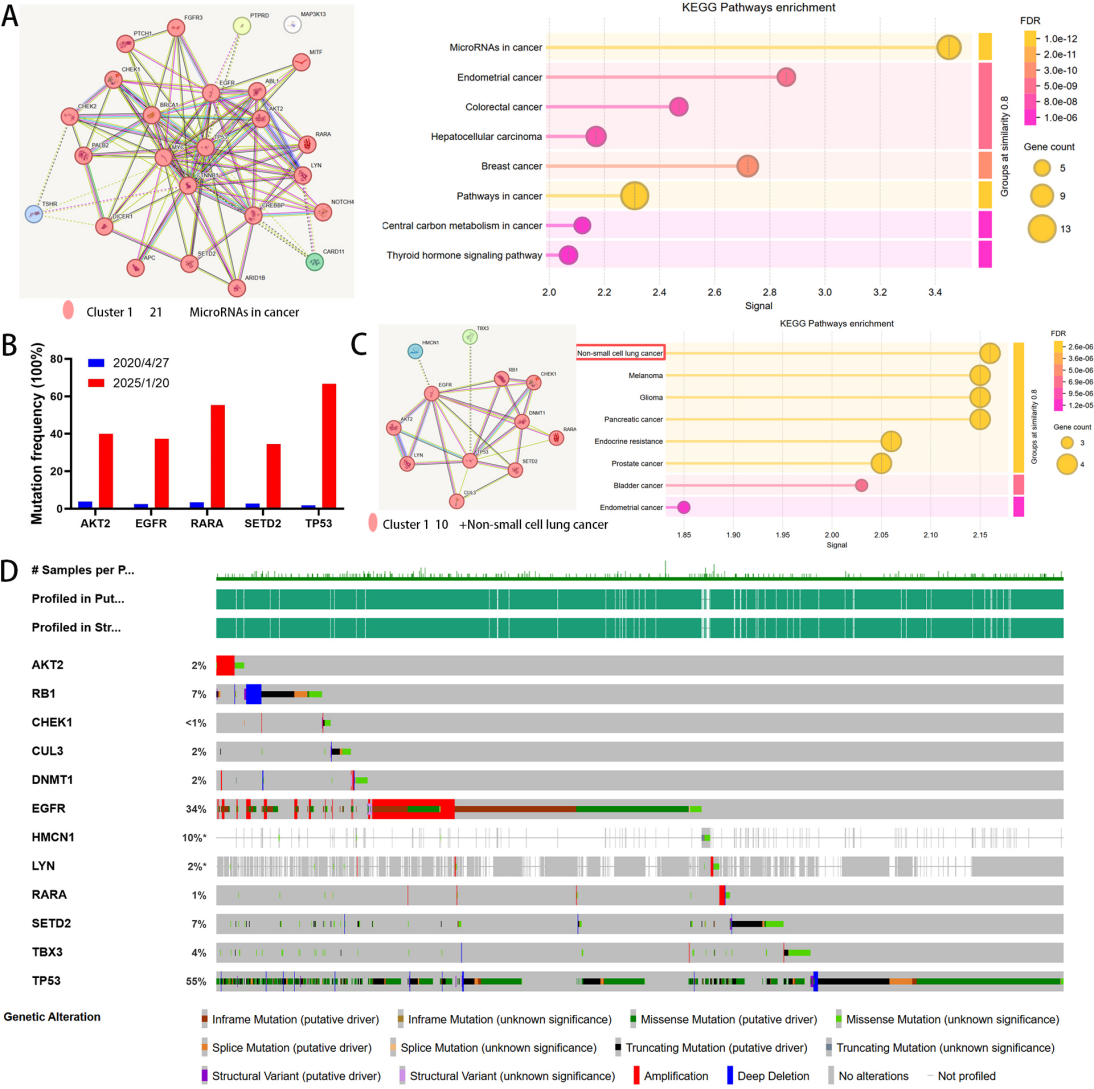
Molecular profiling of genetic alterations. **(A)** Protein-protein interaction network and top 8 enriched KEGG pathways of 25 genes mutated before initial treatment (analyzed via STRING). **(B)** Comparison of variant allele frequencies for 5 key driver genes before gefitinib treatment and after resistance to almonertinib. **(C)** Protein interaction network and top 8 KEGG pathways of 12 co-mutated or loss-of-function genes detected in malignant pleural effusion after almonertinib resistance (STRING analysis). **(D)** Mutation spectrum and frequency of the 12 altered genes within a LUAD cohort from the cBioPortal database (13 studies; n=6,229).

Bioinformatic analysis revealed the 25 initial alterations formed a closely interrelated network by STRING database (53) (**Figure 2A**) and were enriched in key cancer pathways (e.g., “MicroRNAs in cancer,” “Pathways in cancer”), underscoring their oncogenic relevance. Two separate liquid biopsies derived from peripheral blood before initial treatment and after gefitinib resistance showed no detectable mutations, suggesting that circulating tumor DNA from peripheral blood has lower sensitivity compared to testing of primary tumor tissue or pleural effusion cells. This is consistent with previous research findings (54).

Longitudinal genomic profiling revealed dynamic clonal evolution under therapeutic pressure, exemplified by the acquisition of an RB1 copy number loss and a substantial increase in the variant allele frequencies of key driver mutations (e.g., in AKT2, EGFR, RARA, SETD2, and TP53), alongside a decrease in TMB (**Figure 2B**). Notably, no on-target (e.g., T790M, C797X) or common off-target (e.g., MET amplification, ERBB2 amplification) resistance mechanisms were identified (13). The emergence of an RB1 deletion, a known risk factor for EGFR-TKI resistance (55), suggests that resistance may have been mediated through bypass signaling pathways rather than primary EGFR-dependent mechanisms. Previous studies have demonstrated that co-inactivation of tumor suppressor genes, such as TP53 and RB1, significantly shortens both PFS and OS in EGFR-TKI-treated patients (56). Although co-occurring RB1 and TP53 alterations are classically associated with small cell lung cancer (SCLC) transformation (13, 57), immunohistochemical analysis of the pleural effusion cytology in this case confirmed metastatic adenocarcinoma. Thus, the genomic profile derived from the MPE cells must be interpreted with caution regarding this potential transition. In addition to RB1 loss, new mutations emerged in CHEK1, CUL3, DNMT1, HMCN1, and TBX3. The spectrum of mutations acquired after almonertinib resistance formed a highly interconnected protein network, enriched in key pathways relevant to NSCLC and other malignancies (**Figure 2C**).

Interrogation of the cBioPortal database (58) (https://www.cbioportal.org/) (across 13 LUAD studies, n=6,229) revealed no identical genetic alteration profile, and 58.3% (7/12) of the assessed alterations occurred at a population frequency of less than 5% (**Figure 2D**), highlighting the exceptional molecular complexity and individualized nature of treatment resistance in this case. Emerging evidence implicates CHEK1, CUL3, and DNMT1 dysregulation in the progression of EGFR-mutant NSCLC (59–61). Mechanistically, DNMT1 overexpression can activate NF-κB to drive EGFR-TKI resistance, an effect reversible upon DNMT1 inhibition (60). CHEK1 dysfunction, as a DNA damage repair gene, is frequently linked to poor prognosis in NSCLC (62). While CUL3 mutations may compromise its tumor-suppressor function and promote progression (63), the roles of HMCN1 and TBX3 are less defined. Studies in breast and renal cell carcinomas suggest HMCN1 mutations may influence energy metabolism and anti-tumor immunity, though these associations await functional validation (64, 65). TBX3 acts as an oncogene; its overexpression or mutation enhances activity to promote proliferation, angiogenesis, and evasion of apoptosis and senescence (66), and is associated with progression or short-lived response to first-line immunotherapy in metastatic non-squamous NSCLC (67). Despite these insights, the specific contributions of HMCN1 and TBX3 to TKI resistance remain largely unexplored, warranting dedicated mechanistic investigation.

However, that ivonescimab-based therapy still elicited a clinical response in this high-risk molecular context is therefore particularly notable. To our knowledge, this is the first detailed report of successful ivonescimab application—both as monotherapy and in combination with chemotherapy—in a patient with *de novo* MPE and a profoundly complex genomic background following sequential EGFR-TKI failure. Evidence suggests that TP53 mutations can promote the upregulation of VEGF-A expression in NSCLC (68). Furthermore, the altered activity of key tumor suppressor genes including TP53, Rb1, CUL3, and CHEK1, has been associated with elevated PD-L1 expression (69–72). Given this interconnected dysregulation, the dual-targeted mechanism of ivonescimab may potentially reverse the immunosuppressive effects driven by the inactivation of these tumor suppressor genes. Our findings advocate for comprehensive molecular profiling of MPE-derived cells (including NGS and PD-L1 testing) to guide therapeutic decisions in EGFR-TKI-resistant LUAD. Ivonescimab, by simultaneously targeting the tumor vasculature and immune microenvironment, represents a compelling therapeutic strategy for this challenging patient population. Larger prospective clinical trials are urgently needed to validate the efficacy of ivonescimab in this molecularly defined subset.

This single-case report is limited in its generalizability, as the findings are derived from an individual patient experience. A key consideration is the treatment sequence. Although the patient met the eligibility criteria for the phase 3 HARMONi-A trial, which established the superiority of ivonescimab combined with pemetrexed and carboplatin over chemotherapy alone in this setting (49, 50), he declined combination chemotherapy due to profound concerns regarding toxicity—a common challenge in real-world practice. Given the high PD-L1 expression observed in his MPE and the proven efficacy of ivonescimab monotherapy in PD-L1-positive NSCLC (25), he opted for ivonescimab monotherapy. This deviation from the standard protocol precludes direct efficacy comparisons but uniquely demonstrates the standalone activity of dual PD-1/VEGF blockade, which conferred nearly five months of disease control in this molecularly complex case. We acknowledge that upfront combination therapy might have yielded a more robust initial response. This case underscores the tension between protocol-driven care and patient-centered decision-making. Furthermore, this case supports the use of ivonescimab plus pemetrexed as a viable sequential strategy after monotherapy progression, demonstrating a manageable safety profile and sustained clinical benefit.

Second, the retrospective design of this report made it unfeasible to prospectively collect critical immunophenotyping data from the malignant effusion. As a result, detailed information on immune cell subset distribution and cytokine milieu before and after treatment is unavailable. This gap precludes a definitive assessment of the drug’s effect on the local tumor microenvironment and remains a central uncertainty in our mechanistic interpretation.

Third, the diagnosis of metastatic lung adenocarcinoma was unequivocally based on cytology and IHC of the malignant effusion. Despite this, the co-inactivation of RB1 and TP53—a genomic hallmark highly associated with small cell transformation—introduces a note of diagnostic uncertainty. While our diagnostic methods are standard, they cannot definitively exclude the possibility of an occult or focal small cell component that was not sampled in the effusion. An image-guided re-biopsy, though precluded in this case by the primary lesion’s size and location, would have been the only way to fully resolve this genomic ambiguity.

Finally, as with all single-case reports, the findings are inherently preliminary and hypothesis-generating. They require validation in larger, prospective cohorts but offer valuable early insight into a potential therapeutic strategy for this molecularly defined, high-risk population.

In conclusion, this case offers two critical insights for managing EGFR-mutant lung cancer with MPE and complex resistance within the continuously changing treatment landscape (14). First, it underscores the imperative for repeated molecular profiling at progression, preferably using MPE-derived samples rather than plasma, to identify non-canonical resistance mechanisms and assess PD-L1 status. This precision approach is paramount in the current era of immuno-oncology (73), where therapeutic strategies are increasingly biomarker-driven. Second, it contributes preliminary yet promising evidence that ivonescimab—a bispecific antibody targeting both PD-1 and VEGF—is associated with clinically meaningful disease control in this high-risk population, even in a monotherapy setting. This finding aligns with the growing importance of bispecific antibodies in NSCLC, as evidenced by their role in redefining frontline standards for classic EGFR mutations (74) and addressing challenging subgroups with rare co-mutations (75). When the standard ivonescimab-chemotherapy combination is declined, monotherapy may represent a viable alternative. These findings highlight the potential therapeutic value of ivonescimab in this context and call for its formal evaluation in prospective, biomarker-defined cohorts.

## Data Availability

The original contributions presented in the study are included in the article/**Supplementary Material**. Further inquiries can be directed to the corresponding authors.
