# Supplementary material for: Case Report: Ivonescimab in EGFR-mutant lung cancer with baseline malignant pleural effusion and acquired complex resistance
Source: Front Immunol. 2025 Dec 17;16:1725067. doi: 10.3389/fimmu.2025.1725067 (PMC12753922; doi:10.3389/fimmu.2025.1725067)
Supplement: Supplementary file 1 [file Table1.docx]

Supplemental Table 1. Abnormal gene names, mutation sites, and mutation frequencies detected by three high-throughput gene sequencing runs.

| Reporting Time | Specimen Type | Gene name | Mutation type | Mutation frequency | TMB |
| --- | --- | --- | --- | --- | --- |
| 2020-04-27 | FFPE tissue block (from TBLB) | EGFR | p.E746_A750delELREA | 18.92% | 11.57 mut/Mb (High) |
|  |  | ABL1 | c.598C>T p.H200Y | 15.78% |  |
|  |  | AKT2 | c.1101_1102delinsAA p.R368S | 25.26% |  |
|  |  | APC | c.5745G>C p.K1915N | 7.75% |  |
|  |  | ARID1B | c.4945G>T p.A1649S | 6.62% |  |
|  |  |  | c.5740C>T p.R1914W | 17.29% |  |
|  |  | CARD11 | c.1595C>T p.T532M | 6.03% |  |
|  |  | CHEK1 | c.16G>A p.V6M | 14.94% |  |
|  |  | CHEK2 | c.886_895dupGATTATTATA p.I299Rfs*16 | 10.44% |  |
|  |  | CREBBP | c.833A>C p.Q278P | 8.7% |  |
|  |  | CTNNB1 | c.1052C>G p.S351C | 4.7% |  |
|  |  | DICER1 | c.2026C>G p.R676G | 2.44% |  |
|  |  | FGFR3 | c.1349C>T p.T450M | 10.91% |  |
|  |  | LYN | c.1472A>T p.D491V | 8.58% |  |
|  |  | MAP3K13 | c.434T>C p.I145T | 22.39% |  |
|  |  | MITF | c.1363C>A p.L455I | 10.71% |  |
|  |  | MYC | Amplification | n = 3.49 |  |
|  |  | NOTCH4 | c.1748G>A p.G583E | 5.57% |  |
|  |  | PALB2 | c.1054G>C p.E352Q | 7.9% |  |
|  |  |  | c.3251C>G p.S1084W | 4.2% |  |
|  |  | PTCH1 | c.3839C>T p.S1280L | 12.31% |  |
|  |  | PTPRD | c.2158G>A p.V720I | 4.42% |  |
|  |  | RARA | c.847_861del15 p.T283_S287delTMTFS | 28.6% |  |
|  |  | SETD2 | c.1094A>C p.K365T | 18.09% |  |
|  |  | TP53 | c.541C>T p.R181C | 30.27% |  |
|  |  |  | c.637C>T p.R213* | 10.89% |  |
|  |  | TSHR | c.915T>A p.S305R | 14.2% |  |
|  |  | BRCA1 | p.L1086Gfs*5 | 8.28% |  |
|  | Cell pellet from pleural effusion | EGFR | p.E746_A75  0delELREA | 2.51% |  |
|  |  | AKT2 | c.1101_1102delinsAA  p.R368S | 3.8% |  |
|  |  | LYN | c.1472A>T p.D491V | 0.58% |  |
|  |  | TP53 | c.541C>T p.R181C | 1.84% |  |
|  |  | RARA | c.847_861del15 p.T283_S287delTMTFS | 3.52% |  |
|  |  | SETD2 | c.1094A>C p.K365T | 2.81% |  |
|  | Peripheral whole blood | NA | NA | NA |  |
| 2023-04-21 | Peripheral whole blood  (ctDNA:5%) | NA | NA | NA | < 1 mut/Mb (Low) |
| 2025-01-20 | Cell pellet from pleural effusion | AKT2 | NM_001626.6 c.1101_1102delinsAA  p.R368S | 40.05% | 8.72 mut/Mb (Low) |
|  |  | RB1 | Copy number loss | n = 1.17 |  |
|  |  | CHEK1 | NM_001114121.2 c.16G>A p.V6M | 25.14% |  |
|  |  | CUL3 | NM_003590.5c.514G>A p.E172K | 10.89% |  |
|  |  |  | NM_003590.5 c.512G>A p.R171K | 10.88% |  |
|  |  | DNMT1 | NM_001130823.3 c.2914G>A p.V972M | 7.15% |  |
|  |  | EGFR | p.E746_A750delELREA | 37.3% |  |
|  |  | HMCN1 | NM_031935.3 c.1764G>C p.E588D | 10.57% |  |
|  |  |  | NM_031935.3 c.1852C>T p.P618S | 9.29% |  |
|  |  | LYN | NM_002350.4 c.1472A>T p.D491V | 10.73% |  |
|  |  | RARA | NM_000964.4 c.847_861del15  p.T283_S287delTMTFS | 55.46% |  |
|  |  | SETD2 | NM_014159.7 c.1094A>C p.K365T | 34.59% |  |
|  |  | TBX3 | NM_016569.4 c.1673C>T p.S558F | 2.76% |  |
|  |  | TP53 | p.R181C | 66.73% |  |
