# Supplementary material for: Case Report: Ivonescimab in EGFR-mutant lung cancer with baseline malignant pleural effusion and acquired complex resistance
Source: Front Immunol. 2025 Dec 17;16:1725067. doi: 10.3389/fimmu.2025.1725067 (PMC12753922; doi:10.3389/fimmu.2025.1725067)
Supplement: Supplementary file 2 [file DataSheet1.pdf]

CARE case report guidelines

CARE Checklist of information to include when writing a case report

(cc) BY-NC-ND

Topic	Item	Checklist item description	Reported on Line
**Title**	1	The diagnosis or intervention of primary focus followed by the words "case report"	Line #1 (Title Page)
**Key Words**	2	2 to 5 key words that identify diagnoses or interventions in this case report, including "case report"	Line #56-57(Keywords)
**Abstract (no references)**	3a	Introduction: What is unique about this case and what does it add to the scientific literature?	Lines #37-39(Abstract, Background)
	3b	Main symptoms and/or important clinical findings	Lines #40-48(Abstract, Case Presentation)
	3c	The main diagnoses, therapeutic interventions, and outcomes	Lines #48-50(Abstract, Case Presentation)
	3d	Conclusion—What is the main "take-away" lesson(s) from this case?	Lines #51-55(Abstract, Conclusionn)
**Introduction**	4	One or two paragraphs summarizing why this case is unique (**may include references**)	Lines #60-110(Introduction, paragraphs 1-5)
**Patient Information**	5a	De-identified patient specific information.	Lines #112(Case report, paragraphs 1)
	5b	Primary concerns and symptoms of the patient.	Lines #112-113(Case report, paragraphs 1)
	5c	Medical, family, and psycho-social history including relevant genetic information	Not Applicable
	5d	Relevant past interventions with outcomes	Lines #113-122(Case report, paragraphs 1)
**Clinical Findings**	6	Describe significant physical examination (PE) and important clinical findings.	Lines #113-121(Case report, paragraphs 1)
**Timeline**	7	Historical and current information from this episode of care organized as a timeline	Lines #124 (Case report, Figure 1A)
**Diagnostic Assessment**	8a	Diagnostic testing (such as PE, laboratory testing, imaging, surveys).	Lines #115-121 , #130-134 (Case report, paragraphs 1&3)
	8b	Diagnostic challenges (such as access to testing, financial, or cultural)	Lines #193-208 (Discussion, paragraphs 5)
	8c	Diagnosis (including other diagnoses considered)	Lines #193-208 (Discussion, paragraphs 5)
	8d	Prognosis (such as staging in oncology) where applicable	Lines #122 (Case report, paragraphs 1)
**Therapeutic Intervention**	9a	Types of therapeutic intervention (such as pharmacologic, surgical, preventive, self-care)	Lines #123-144 (Case report, paragraphs 2-3)
	9b	Administration of therapeutic intervention (such as dosage, strength, duration)	Lines #123-144 (Case report, paragraphs 2-3)
	9c	Changes in therapeutic intervention (with rationale)	Lines #123-150 (Case report, paragraphs 2-4)
**Follow-up and Outcomes**	10a	Clinician and patient-assessed outcomes (if available)	Lines #123-144 (Case report, paragraphs 2- 3)
	10b	Important follow-up diagnostic and other test results	Lines #123-150 (Case report, paragraphs 2-4)
	10c	Intervention adherence and tolerability (How was this assessed?)	Lines #145-150 (Case report, paragraphs 4)
	10d	Adverse and unanticipated events	Lines #145-150 (Case report, paragraphs 4)
**Discussion**	11a	A scientific discussion of the strengths AND limitations associated with this case report	Lines #152-233 (Discussion)
	11b	Discussion of the relevant medical literature **with references**.	Lines #152-233 (Discussion)
	11c	The scientific rationale for any conclusions (including assessment of possible causes)	Lines #152-233 (Discussion)
	11d	The primary "take-away" lessons of this case report (without references) in a one paragraph conclusion	Lines #226-233 (Discussion, paragraphs 4)
**Patient Perspective**	12	The patient should share their perspective in one to two paragraphs on the treatment(s) they received	Not Applicable
**Informed Consent**	13	Did the patient give informed consent? Please provide if requested	Yes ☑ No ☐
